# Low-Dose Decitabine-Based Chemoimmunotherapy for Patients with Refractory Advanced Solid Tumors: A Phase I/II Report

**DOI:** 10.1155/2014/371087

**Published:** 2014-05-21

**Authors:** Hui Fan, Xuechun Lu, Xiaohui Wang, Yang Liu, Bo Guo, Yan Zhang, Wenying Zhang, Jing Nie, Kaichao Feng, Meixia Chen, Yajing Zhang, Yao Wang, Fengxia Shi, Xiaobing Fu, Hongli Zhu, Weidong Han

**Affiliations:** ^1^Department of Bio-Therapy, College of Life Sciences, Chinese PLA General Hospital, 28 Fuxing Road, Haidian District, Beijing 100853, China; ^2^Department of Geriatric Hematology, Chinese PLA General Hospital, 28 Fuxing Road, Haidian District, Beijing 100853, China; ^3^Department of Immunology, Institute of Basic Medicine, Medical College of PLA, Chinese PLA General Hospital, Beijing 100853, China

## Abstract

Aberrant DNA methylation is one of the main drivers of tumor initiation and progression. The reversibility of methylation modulation makes it an attractive target for novel anticancer therapies. Clinical studies have demonstrated that high-dose decitabine, a hypomethylating agent, results in some clinical benefits in patients with refractory advanced tumors; however, they are extremely toxic. Low doses of decitabine minimize toxicity while potentially improving the targeted effects of DNA hypomethylation. Based on these mechanisms, low-dose decitabine combined with chemoimmunotherapy may be a new treatment option for patients with refractory advanced tumors. We proposed the regimen of low-dose decitabine-based chemoimmunotherapy for patients with refractory advanced solid tumors. A favorable adverse event profile was observed in our trial that was highlighted by the finding that most of these adverse events were grades 1-2. Besides, the activity of our cohort was optimistic and the clinical benefit rate was up to 60%, and the median PFS was prolonged compared with PFS to previous treatment. We also identified a significant correlation between the PFS to previous treatment and clinical response. The low-dose DAC decitabine-based chemoimmunotherapy might be a promising protocol for improving the specificity and efficiency of patients with refractory advanced solid tumors. This trial is registered in the ClinicalTrials.gov database (identifier NCT01799083).

## 1. Introduction

Traditional therapies, including chemotherapy, radiotherapy, and surgery, have been proven to be helpful in the management of numerous solid and hematologic cancers. However, most patients eventually develop resistance to these treatments, and over 90% of cancer patients die from refractory and metastatic disease [1, 2]. Given the frequent failure of conventional salvage therapy in the treatment of refractory and relapsed tumors, innovative strategies are urgently needed.

Recently, it has become clear that tumors can be driven by patterns of altered gene expression that are mediated by mechanisms of epigenetic regulation, such as DNA methylation. DNA methylation typically occurs at the 5′-position of the cytosine ring within cytosine-phosphate-guanine (CpG) dinucleotides, and DNA methyltransferases (DNMTs) catalyze this reaction [3, 4]. In normal cells, CpG islands of tumor suppressors are usually unmethylated; however, hypermethylation of CpG promoters occurs frequently in tumors [5, 6]. DNA demethylation has the potential to reverse promoter hypermethylation in tumor cells and lead to the reexpression of aberrantly silenced genes, such as tumor suppressor genes (TSGs) of p16 and p15 [7] and cancer testis antigens (CTA) of MAGEA-1 and MAGEA-3 [8], and to induce the sensitivity of tumor cell to anticancer agents. It has been demonstrated that DNA demethylation can be an effective therapy for myelodysplastic syndrome, which is characterized by global promoter hypermethylation [9, 10]. Decitabine (DAC) is a DNA demethylating agent [11] that was initially tested as a cytotoxic chemotherapeutic agent that is incorporated into the RNA at high doses. Approximately 20 years later, DAC was discovered to possess DNA demethylating activity at low doses when incorporated into the DNA. Decitabine has been reported to inactivate DNA methyltransferases (DNMTs) by forming a covalent complex at CpG methylation sites. Off-target effects can occur with high doses DAC, and these effects can include triggering DNA damage and cell cycle alterations that are immediately cytotoxic [12].

Additionally, preclinical data suggest that DAC can significantly reverse the expressions of genes that are differentially regulated at the relapse stage, and some of these genes may play a role in chemoresistance [13]. DAC has also been proposed to possess immunomodulatory activity that is mediated by the restoration of the proper expression of immune receptors and their ligands. The epigenetic remodeling induced by DAC has been suggested to enhance tumor immunogenicity and tumor susceptibility to immune destruction by upregulating the expression of tumor antigens and major histocompatibility complex (MHC) class I in cancers [14].

Clinical studies have demonstrated that high dose DAC treatment regimens result in some clinical benefits in patients with malignancies; however, these regimens have been shown to be extremely toxic due to the poor hematologic status of these patients and may even cause death [15]. Low-dose DAC minimizes toxicity while potentially retaining the inhibition of the activities of DNA methyltransferases via incorporation into the DNA [16]. The lowest reported total dose of decitabine that has been used to treat a solid tumor is 50 mg/m^2^, but this dose was accompanied by various adverse events [17]. A study of natural killer cell showed hypomethylation due to low-dose decitabine (0.02–2.5 *μ*M) and cytotoxicity and increased methylation at higher doses (>2.5 *μ*M) [18]. Based on these observations and the short half-life of decitabine and its absolute requirement of DNA synthesis for activity, we conducted a phase I/II trial using low-dose decitabine (7 mg/m^2^) administered over five consecutive days with the intent of reducing the toxicity and improving the targeted effects on DNA hypomethylation in patients with refractory advanced solid tumors. Additionally, we used regimens of low-dose DAC combined with chemotherapy or adoptive immunotherapy to determine whether low-dose DAC could functionally restore chemosensitivity and enhance the efficiency of adoptive immunotherapy in refractory advanced solid tumors.

## 2. Patients and Methods

### 2.1. Patients

Eligible patients were required to have a histological/cytological diagnosis of refractory advanced solid tumors and at least one site of radio graphically measurable disease ≥2 cm in the largest dimension by traditional computed tomography (CT) or ≥1 cm in the largest dimension by spiral CT. Additional eligibility criteria were as follows: patients who had received at least three weeks of effective first-line combination chemotherapy regimens, radiotherapy, major surgery, or any other investigational anticancer therapy; patients who had progressive disease after the most recent treatment regimen and had recovered from previous toxic effects; and patients who had performance statuses of two or less on the Eastern Cooperative Oncology Group (ECOG) performance scale and adequate organ function. The eligible participants also had to have a life expectancy of at least six months and an adequate hematologic profile (white blood cells: 3.0 × 10^9^/L; platelets: 100 × 10^9^/L; and hemoglobin: 110 g/L). Informed consent was obtained from the patients or a legal guardian prior to enrollment.

The exclusion criteria included pregnant or lactating women, patients with myocardial infarction, unstable angina within six months, and significant cardiovascular disease, patients who suffered from internal organ injuries of the liver, heart, or kidney, and those with signs of internal bleeding.

### 2.2. Preparation of Cytokine Induced Killer (CIK) Cells

All technicians who performed the CIK cell culture and quality control were healthy and received training in good manufacturing practices. A total of 50 mL of venous blood were collected into evacuated tubes containing heparin, and peripheral blood mononuclear cells (PBMCs) were subsequently isolated by Ficoll-Hypaque density-gradient centrifugation. The PBMCs were grown in serum-free medium, and the cell densities were adjusted to meet predetermined criteria. The expansion and induction of cultured PBMCs into CIK cells were mainly performed as we have previously described [19].

### 2.3. Study Design and Treatment Protocol

**Figure 1:**
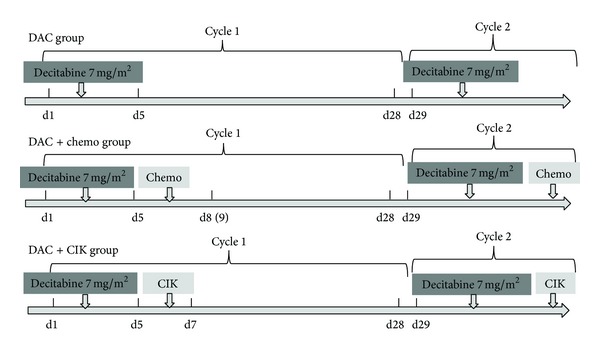
Study design. Chemo: chemotherapy; CIK: cytokine induced killer cells.

This was a single-center, open-label, double-blind, and prospective investigator-initiated phase I/II study conducted at the Chinese PLA General Hospital (ClinicalTrials.gov: NCT01799083). This study was an interim analysis of our registered phase I/II clinical trial of low-dose DAC, entitled “Low Dose Decitabine-Based Therapy in Patients with Refractory and/or Chemotherapy Resistant Solid Tumors or B Cell Lymphomas.” The present study was undertaken in accordance with principles of good clinical practice. The patients were enrolled between February 2012 and August 2013 and were categorized into the following three groups, according to clinical status and the radiographic outcomes (Figure 1): the DAC group (these patients were given 7 mg/m² decitabine by intravenous push for five days of each 28-day treatment cycle), the DAC combined with chemotherapy group (these patients were given 7 mg/m² decitabine for five days followed by chemotherapies that were administered primarily in the same manner as their previous ineffective regimens in 28-day cycles), and the DAC combined with CIK cells group (these patients were given 7 mg/m² decitabine for five days followed by CIK cells at 1.0–5.0 × 10^9^/L for two days in 28-day cycle). Decitabine (DacoGen, Pharmachemie BV) was stored as a stable freeze-dried powder in 50 mL vials, reconstituted in 10 mL of sterile water for injection, and diluted to a final volume of 25 mL. The chemotherapy regimens included the following etoposide (VP-16): pemetrexed disodium, irinotecan, 5-fluorouracil, and R-COP (cyclophosphamide, vindesine, prednisone, and rituximab); R-CHOP (rituximab, cyclophosphamide, adriamycin, vincristine, and prednisone); CHOP (cyclophosphamide, adriamycin, vincristine, and prednisone); COP (cyclophosphamide, vindesine, and prednisone); R-GEMOX (rituximab, gemcitabine, and oxaliplatin); and so forth. There were no protocol-specific premedications. All patients underwent a complete medical interview and a physical examination that included a blood profile and a CT of the disease lesions. The patients were restaged by CT every two cycles.

For patients with any grade 4 hematological or other nonhematological adverse events that were considered to be related to decitabine, chemotherapy, or adoptive immunotherapy, the treatment was discontinued for two weeks to resolve the event to below grade 1 or to baseline. Additionally, treatment was delayed if the patient did not recover from toxicity within the following two weeks. If more than two weeks were required for a toxic effect to resolve, the patient was removed from the study because of that adverse event. Additionally, the treatments for the patients whose white blood cell counts were less than 3.0 × 10^9^/L or platelet count was less than 100 × 10^9^/L were suspended. Patients with evidence of progressive disease at the first tumor assessment were allowed to continue to receive decitabine unless the patient's health was declining rapidly. Progressive disease was confirmed by two scans that were performed at least four weeks apart. Toxicity and clinical efficiency were assessed after at least three cycles of treatment because the hypomethylating activity of decitabine is replication dependent (decitabine requires several cell divisions to complete the demethylation of each DNA strand).

### 2.4. Assessment of Efficiency and Adverse Events

The primary endpoint of the current trial was the safety of the sequential use of low-dose DAC-based chemoimmunotherapy. Toxicity assessments were performed at each cycle during the therapy and were graded according to the National Cancer Institute Common Terminology Criteria for adverse events (CTCAE v3.0).

The secondary outcomes were as follows. (1) The feasibility of low-dose DAC-based chemoimmunotherapy was evaluated. To assess the disease response and duration of progression-free survival (PFS: defined as the time from randomization to disease progression or death, whichever occurred first), CT assessment was performed after two cycles. The feasibility evaluation was carried out using the Response Evaluation Criteria in Solid Tumors (RECIST 1.0). (2) The target modulation activities of DAC in reducing DNA demethylation and reexpression of epigenetically silenced genes in peripheral blood mononuclear cells (PBMCs) were evaluated.

### 2.5. Biological Studies

#### 2.5.1. Blood Collection

Peripheral blood samples (10 mL) were collected from the patients into ethylene-diamine tetraacetic acid (EDTA) vials prior to treatment and on the last day of each cycle. PBMCs were obtained by Ficoll-Hypaque density-gradient centrifugation and were viably cryopreserved in liquid nitrogen for use in subsequent assays.

#### 2.5.2. Cell Lines and Drug Treatment Conditions

The human hepatocellular carcinoma HepG2 cell line was obtained from the American Type Culture Collection. DAC was purchased from Sigma Chemical Company. The effects of DAC treatment on gene expression were determined after exposure to 10 nM DAC for 72 hours.

#### 2.5.3. RNA Isolation and qRT-PCR

RNA isolation was performed using Trizol Reagent (Invitrogen, Carlsbad, CA) according to the manufacturer's instructions. Gene validation was performed by quantitative real-time polymerase chain reaction (qRT-PCR). Initially, cDNA was synthesized from 1 *μ*g of total RNA using a RevertAid First Stand cDNA Synthesis Kit (Thermo Fisher, USA) in a total volume of 20 *μ*L. Reverse transcription was performed at 42°C for 60 min. Real-time PCR was performed using a SYBR Green PCR Mix Kit (Toyobo) according to the manufacture's protocol. The primers for RASSF1A, p16, p15, MAGEA-3, MAGEA-1, BRCA1, and *β*-actin are shown in Table 6(a). The relative expressions of RASSF1A, p16, p15, MAGEA-3, MAGEA-1, and BRCA1 were normalized to the internal control of *β*-actin with the 2^−ΔΔCt^ cycle threshold method.

#### 2.5.4. DNA Extraction and Methylation Analysis

Genomic DNA was extracted from peripheral blood according to the manufacturer's instructions using the AxyPrep Blood Genomic DNA Miniprep Kit (Axygen). The sodium bisulfite conversion of DNA was modified using the CpG DNA Modification Kit (Millipore, Billerica, MA) according to the manufacturer's instructions. After the chemical modification of DNA, PCR analysis was carried out using primers that were designed specifically to utilize the sequence differences between the methylated and unmethylated DNA that result from bisulfate treatment. All presented data reached preset acceptance criteria. A bisulfite modification check was performed to confirm that the DNA had been satisfactorily modified. The primer sequences and methylation-specific PCR conditions are detailed in Table 6(b).

### 2.6. Statistical Analyses

The analyses of the demographic characteristics, such as age, gender, and baseline characteristics, were descriptive. The hypothesis of the current trial was that the administration of low-dose DAC-based chemoimmunotherapy would improve the PFSs of the patients relative to their previous treatments. We used the Swim plot method to estimate the PFSs and regarded *P* values below 0.05 as significant. We used IBM-SPSS version 20.0 for all statistical analyses. All patients were included in the analyses.

To analyze the changes in the expression of the RASSF1A, p16, p15, MAGEA-3, MAGEA-1, and BRCA1 genes, the data are shown as the mean ± S.D. Statistical comparisons between experimental groups were performed using Student's *t*-tests and one-way analyses of variance, and a two-tailed *P* value <0.05 was considered significant.

## 3. Results

### 3.1. Patient Characteristics

**Figure 2:**
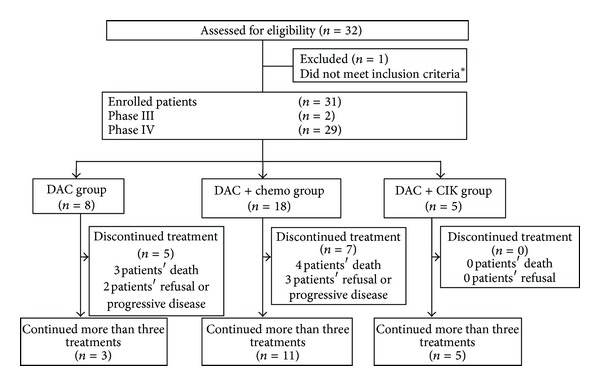
Trial profile. (∗) Reasons for not meeting the inclusion criteria: patient had a massive hemorrhage (*n* = 1). CIK: cytokine induced killer cells. DAC + Chemo: DAC combined with chemotherapy group; DAC + CIK: DAC combined with CIK group.

A total of 32 patients with 14 different malignancies (gastric cardia adenocarcinoma, colorectal adenocarcinoma, hepatocellular carcinoma, intrahepatic bile ducts adenocarcinoma, alveolar carcinoma, malignant pleural tumors, esophageal adenocarcinoma, non-Hodgkin's lymphoma, Hodgkin's lymphoma, lung adenocarcinoma, cervical squamous cell carcinomas, ovary serous papillary cystadenocarcinoma, tubal serous adenocarcinoma, and pancreatic cancer) were included in the present study, and one patient was excluded from the analyses due to massive hemorrhage. In these subjects, three were at level III, and 28 were at level IV. There were 10 (32.26%) women and 21 (67.74%) men. The average age was 58.8 years (range: 28 to 84 years). The majority of patients had ECOG PSs of 0–2. The baseline characteristics of the patients are summarized in Table 1. Among these patients, eight were assigned to receive low-dose DAC, eighteen patients were assigned to receive low-dose DAC combined with chemotherapy, and five patients were assigned to receive low-dose DAC combined with CIK cells (Figures 1 and 2).

The subjects comprised a population of heavily pretreated and advanced-refractory patients with an average of nine previous therapies on average (range: 1–29). The majority of patients had achieved a partial response and disease progression was documented within six months of completing their last previous treatment of chemotherapy or other regimens (Table 2). The reasons for patient discontinuation were death or progressive disease (15 of 31; 48.39%) and patient refusal of the therapy (4 of 31; 12.90%).

### 3.2. Adverse Events

All patients were evaluable for toxicity. The most common adverse events (AEs) are listed in Table 3. Overall, 27 patients (93%) had grade 1-2 treatment-related adverse events, four patients (14%) had grade 3-4 treatment-related adverse events, and none of the 31 patients withdrew from the study because of adverse events. The commonly reported adverse events, irrespective of causality, were neutropenia (52.5%), nausea (12.5%), and fatigue (22.5%). Neutropenia was the most common adverse event associated with decitabine. The patients in the low-dose DAC combined with chemotherapy group experienced more adverse events than the other two groups, particularly in terms of blood and lymphatic system disorders and gastrointestinal symptoms. We noted an increase in grade 3 and grade 4 neutropenia in this group. We also noted increases in nausea, fatigue, and drowsiness in the low-dose DAC combined with chemotherapy group, although most of these symptoms were grades 1-2. No obvious adverse effects were observed in the patients in the low-dose DAC combined with CIK group which were found to have no obvious adverse events.

All of these adverse events were easily managed medically, resolved spontaneously, and generally required no intervention with the exception of symptomatic therapy, including antiemetics or antifebrile or other agents for gastrointestinal side effects. No discontinuations occurred due to concerning decitabine-related toxicity, and no patients died from causes related to treatment.

### 3.3. Efficiency and Clinical Benefits

#### 3.3.1. Low-Dose DAC

Of the eight patients treated with low-dose DAC, five did not complete the treatment for three cycles. Four of them died due to disease progression, and one patient refused the treatment (Figure 2). The remaining three patients were treated for more than three cycles. Among these three patients, one exhibited a partial response (PR) (five months at the end of the follow-up period), one had progressive disease (PD), and the remaining patient discontinued the treatment due to digestive tract hemorrhage (Table 4).

The disease of the patient who achieved a PR was determined to be a malignant pleural tumor. She had previously been treated with nine cycles of pemetrexed, after which she developed disease progression. She began a regimen of low-dose DAC, was restaged after six cycles, and showed PR by RECIST.

Among all patients treated with low-dose DAC, the median PFS was 2.5 months (range: 1–12 months) compared with one month (range: 1–20 months) for the previous treatments. Comparisons of the PFSs revealed that at least two patients had longer PFSs with low-dose DAC than with their previous treatments (Figure 4(a) and Table 5). Although we noted no significant correlation between tumor histology and clinical benefit, we identified a significant correlation between the PFS for previous treatment and the clinical response. For the patient who achieved response, the PFSs of the previous treatments were 20 months. However, for the patient who had progressive disease, the PFSs of the previous treatments were two months. The PFSs for previous treatments for each of the four patients who died were zero months (Figure 4(a)).

#### 3.3.2. Low-Dose DAC in Combination with Chemotherapy

Of the 18 enrolled patients who were treated with low-dose DAC combined with chemotherapy, four patients were treated for less than three cycles. Among these patients, two patients died due to disease progression, and two patients discontinued treatment. Fourteen patients were treated for more than three cycles, one of whom achieved a partial response that persisted for six months, and another patient exhibited a partial response that persisted for three months at the end of the follow-up period. Five patients achieved disease stability after six cycles of treatment (Table 4). The median PFS for the 11 progression-evaluable patients who were treated for six cycles was four months (range: 1–7 months) compared with two months (range: 1–5 months) for the previous treatments.

For disease of the patient who achieved PR, it was was confirmed to be ovary serous papillary carcinoma. She had previously been treated with surgery, topotecan, paclitaxel/carboplatin, paclitaxel/cisplatin, cisplatin/cyclophosphamide, and etoposide, after which she developed disease progression. She underwent a combined regimen of low-dose DAC and paclitaxel/carboplatin for four cycles and exhibited a PR as demonstrated by RECIST scores and a decreased CA125 level.

**Figure 3:**
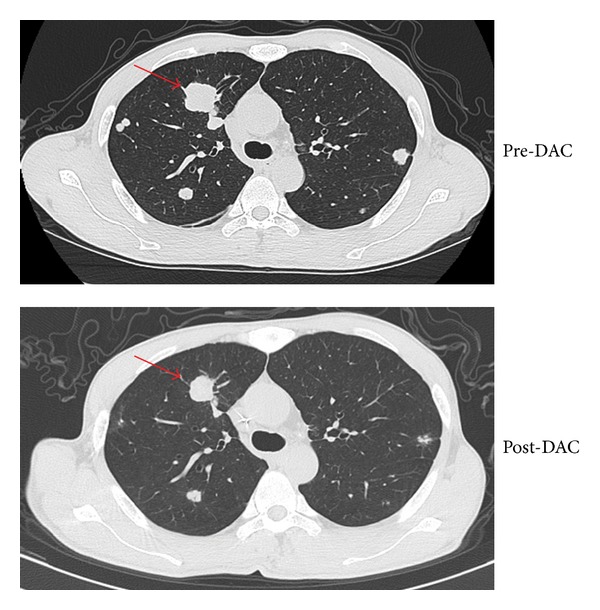
CT image for UPN 20 exhibited a partial response. A CT image showing the specific characteristics of the response of lung lesion following four cycles of decitabine treatment, assessing by the Response Evaluation Criteria in Solid Tumors (RECIST). The red arrows indicate the areas of measurable disease.

The disease of another patient who achieved PR was confirmed to be lung carcinoma. He had previously been heavily treated with surgery, radiation, and gemcitabine/carboplatin for two cycles and gemcitabine/cisplatin for three cycles, after which he developed disease progression. He underwent a combined regimen of low-dose DAC and gemcitabine/carboplatin or gemcitabine/cisplatin for four cycles, and restaging showed PR by RECIST (Figure 3).

**Figure 4:**
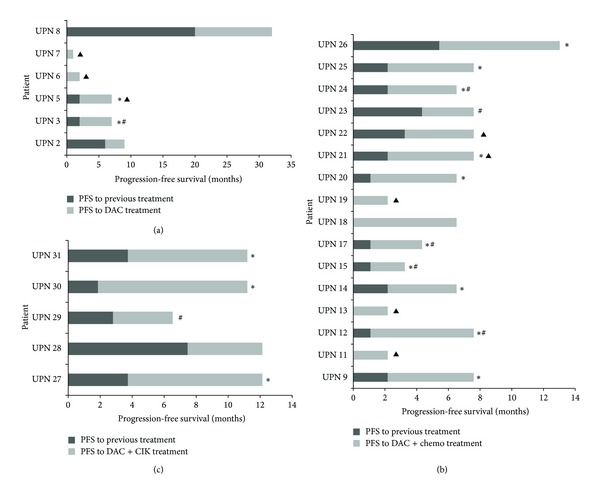
Swim plot showing the increase in progression-free survival (PFS) compared with the patients' previous therapies. The bars represent the progression-free survivals (PFSs) of the DAC (a) and DAC combined with chemo (b) or CIK (c) versus the PFSs following the patients' previous therapies. Four patients had died by the first assessment (due to disease progression) and were therefore not evaluated. ∗ The patients' progression-free survivals (PFSs) were significantly prolonged compared with those of previous therapies; *P* value <0.05 was considered significant. CIK: cytokine induced killer cells. # Patients with disease progression. ▲ Patients who died.

In contrast to the low-dose DAC group, we did not identify a significant correlation between the PFS to previous treatment and the clinical response in the low-dose DAC combined with chemotherapy group. However, we also recognized that, for the patients with progressive disease, the average PFS to previous treatment was only 1.6 months and that for the patients who died was only one month. Additionally, we found that ten patients exhibited statistically significant differences between the PFSs for their current and previous treatments (Figure 4(b) and Table 5). We also found that the efficiency of low-dose DAC combined with chemotherapy might be related to the locations of the tumors. For example, a response may be obtainable if the tumor was located in the ovary or lung, and stable disease was possibly achieved if the tumor was located in lymphoma or tubal; however, this treatment regimen was perhaps ineffective for some digestive system tumors such as gastric and esophageal carcinomas.

#### 3.3.3. Low-Dose DAC in Combination with CIK Cells

Of the five enrolled patients who were treated with low-dose DAC combined with CIK cells, all were treated for more than three cycles. Among these patients, four achieved disease stability persisting for six months (at the end of the follow-up period), and one patient exhibited progressive disease. The median PFS for these five progression-evaluable patients was eight months (range: 4–10 months) compared with four months (range: 2–8 months) for their previous treatments. Compared with their previous therapies, three patients exhibited longer PFSs with low-dose DAC combined with CIK cells, and most of the patients with prolonged PFSs achieved responses (Figure 4(c) and Table 5).

Although we noted no significant response among these five patients, four of them achieved disease stability that persisted for the duration of the disease assessment; therefore, it is possible that these patients would have achieved significant responses during the follow-up time. Additionally, compared with low-dose DAC, low-dose DAC in combination with CIK cells significantly prolonged progression-free survival and improved the efficiency, which suggests that low-dose DAC might play an important role in the immunological regulatory effect of CIK cells.

Similar to the low-dose DAC group, we identified a significant correlation between the PFS to previous treatment and the clinical response among the low-dose DAC combined with CIK group. This correlation showed that, for the patients who achieved disease stability, the average PFS for their previous treatment was 4.5 months (range: 3–8 months). However, for the patients who had progressive diseases, the PFSs for their previous treatments averaged only three months.

Overall, the median PFS for all of the progression-evaluable patients was four months (range: 1–12 months) compared with two months (range: 1–20 months) for their previous treatments. The partial response (PR) and stable disease (SD) rates were 15.79% and 47.37%, respectively.

#### 3.3.4. Biomarker Studies

**Figure 5:**
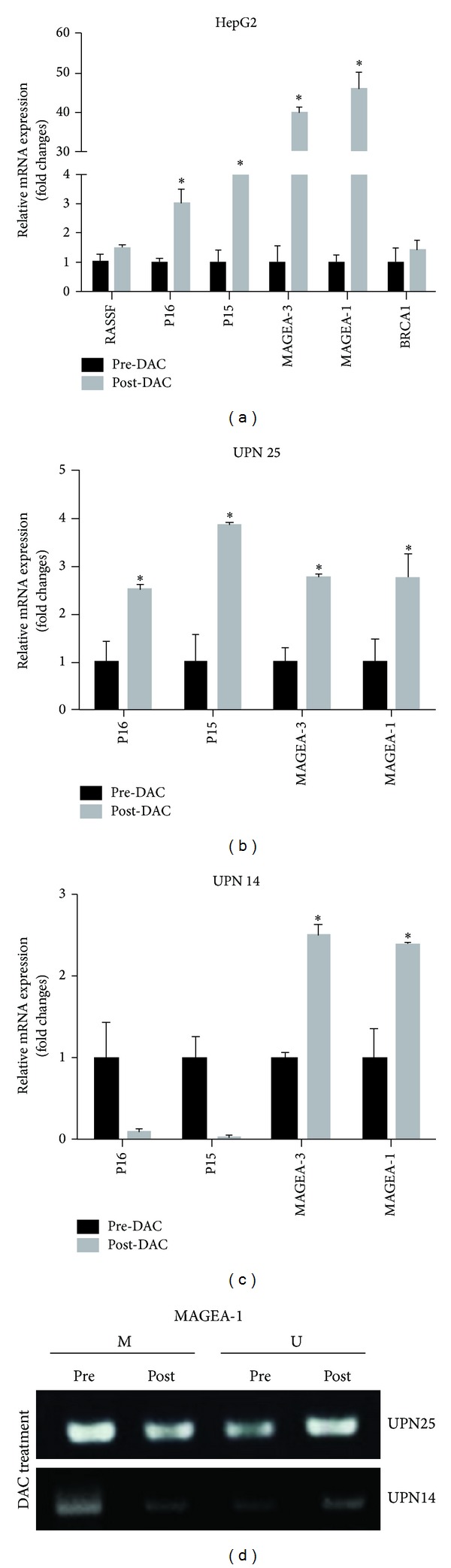
*In vivo* and* in vitro* biological activities of decitabine (DAC) were shown in human hepatocellular carcinoma HepG2 cell line and peripheral blood mononuclear cells (PBMCs). (a) Quantitative RT-PCR analyses of the mRNA levels of RASSF1A, MAGEA-3, MAGEA-1, p16, p15, and BRCA1 expression on human hepatocellular carcinoma HepG2 cell line; the cell lines were treated with 10 nM DAC for 72 h before collecting mRNA for analysis. Compared to untreated control, the mRNA expression levels of MAGEA-3, MAGEA-1, p16, and p15 were augmented significantly in treated cell line. (b, c) Quantitative RT-PCR analyses of the mRNA levels of MAGEA-3, MAGEA-1, p16, and p15 expression in peripheral blood mononuclear cells (PBMCs) from patients who exhibited prolonged disease stabilization following low-dose DAC treatment for the first cycle. Progressive increases in the mRNA expressions of MAGEA-3, MAGEA-1, p16, and p15 were observed in patient UNP 25; in contrast, the p16 and p15 mRNA expressions were reduced in patient UNP 14. (d) Methylation-specific PCR analyses of the changes in MAGEA-1 promoter methylation levels in peripheral blood mononuclear cells (PBMCs) collected from patients UNP 25 and UNP 14, during the first treatment cycle. The levels of MAGEA-1 promoter methylation of patients UNP 14 and UNP 25 were reduced and the levels of MAGEA-1 promoter unmethylation were increased at the same time. M: methylation; U: unmethylation. *P < 0.05, for the significance of the gene expressions differences between the DAC treatment sample and the pre-DAC sample.* Error bars represent standard deviation of the measurements. *

The expressions of the tumor suppressors RASSF1A, p16, p15, and BRCA1 and melanoma antigen gene family-MAGEA-3 and -MAGEA-1 have been reported to undergo epigenetic modulation in a large set of primary human tumors [8, 20]. Our qRT-PCR analyses revealed that the mRNA expression levels of MAGEA-3, MAGEA-1, p16, and p15 were significantly increased in the hepatocellular carcinoma HepG2 cell lines after treatment with 10 nM DAC for 72 h and that the RASSF1A and BRCA1 levels were unchanged (Figure 5(a)). Moreover, we detected the mRNA levels of MAGEA-3, MAGEA-1, p16, and p15 in PBMCs obtained from two patients who exhibited prolonged disease stabilization following treatment with 35 mg/m^2^ DAC. In patient UNP 25, progressive increases in MAGEA-3, MAGEA-1, p16, and p15 mRNA expression levels were observed (Figure 5(b)). In patient UNP 14, MAGEA-3 and MAGEA-1 mRNA expression levels were also increased compared with those at pretreatment, and the p16 and p15 levels were reduced (Figure 5(c)).

We subsequently directly assessed the demethylating effects of DAC in patients using methylation-specific PCR assays. As shown in Figure 5(d), the methylation levels of the MAGEA-1 promoters in the PBMCs from patients UNP 25 and UNP 14 were dramatically reduced and the levels of DNA unmethylation were increased at the same time, and these results correspond to the mRNA expressions shown in Figure 5(d). The data from these patients suggest that low-dose DAC treatment may have reversed the DNA methylation and induced the reexpression of known epigenetically regulated genes both* in vitro* and* in vivo* to contribute to the antitumor activity. Nevertheless, the effects of low-dose DAC-based chemoimmunotherapy varied across the patients for whom we were able to perform biomarker analyses, and this variation accords with clinical observations.

## 4. Discussion

Although it has been shown that epigenetic agents, such as decitabine, can be used as monotherapies for hematologic cancers, accumulating evidence strongly suggests that these agents will be more effective when combined with conventional chemotherapies. Decitabine can activate proapoptotic pathways or inhibit oncogenic signaling cascades, but decitabine does not directly induce the reexpression of caspase-8, which plays an important role in the chemotherapy resistance of tumors; therefore, decitabine may increase the susceptibility of tumor cells to chemotherapy [21–23]. Decitabine may also be used as an immune modulator to sensitize the tumor cells to tumor necrosis factor-related apoptosis-inducing ligand (TRAIL) [24]. Moreover, a preclinical study reported that decitabine improves the immune recognition of tumor cells by both methylation-regulated and nonmethylation-regulated target antigens [25].

In the present study, we first examined the safety of low-dose decitabine-based chemoimmunotherapy in patients with refractory advanced solid tumors. These regimens had safety and tolerability profiles that support further study in refractory solid tumors. We found that all of these regimens had good toxicity profiles, and most of the adverse events were grades 1-2. Myelosuppression is the most common adverse event associated with decitabine [26]. In the current study, 20% of the patients developed grades 3-4 neutropenia, and the majority of these patients were treated with decitabine combined with chemotherapy. Compared with previous reports [17], decitabine combined with chemotherapy did not result in many serious adverse events across the clinical development. Additionally, the most common nonhematologic toxicities resulting from decitabine include mild injection site reaction and nausea [27]. In the present study, the common nonhematologic toxicities were fatigue, nausea, hyperhidrosis, and drowsiness. These adverse events were well tolerated and anticipated for both low-dose DAC and low-dose DAC combined with chemotherapy. However, these nonhematologic toxicities were absent in the patients who received low-dose DAC combined with CIK cells. Furthermore, no patient died from treatment-related adverse events.

We found that the adverse events of patients treated with low-dose DAC combined with chemotherapy were much more severe than those treated with low-dose DAC and low-dose DAC combined with CIK cells. Therefore, we speculated that treatment with low-dose DAC combined with CIK cells, which has minimum cytotoxicity, can be used for frail patients to avoid intervention-related risks. However, low-dose DAC combined with chemotherapy may be a practical alternative in the treatment of well-tolerated patients.

Our study revealed responses across multiple relapsed or refractory advanced cancers in the patients treated with low-dose decitabine-based chemoimmunotherapy. Among these evaluable patients, three partial responses (15.79%) were observed, including two in patients with ovarian carcinoma and lung squamous cell carcinoma who were treated with low-dose DAC combined with chemotherapy and one patient with a malignant pleural tumor who was treated with low-dose DAC. Nine stable disease cases (47.37%) were observed and included patients with hepatocellular carcinoma, gastric cardia adenocarcinoma, and colorectal cancer, and the rate at which patients in the low-dose DAC combined with CIK group achieved a stable disease state was 80%. There were differences in the clinical efficacy that have reported in previous investigations. For example, Stathis et al. [28] and George et al. [29] showed that decitabine in combination with other drugs produced no objective responses in patients with advanced solid tumors and hematologic malignancies. Appleton et al. [30] observed a 40% clinical response rate in patients with solid tumors to the combination of decitabine and carboplatin. We also showed that the increased incidence of the cytotoxicity was significantly increased in the patients who were treated with low-dose DAC combined with chemotherapy; however, this therapy simultaneously improved the clinical responses of patients with refractory advanced tumors.

Although our regimens only achieved partial responses in three patients who were heavily pretreated and had relapsed malignant tumors, the progression-free survivals of these patients were prolonged compared with those achieved with their previous treatments. Moreover, because the hypomethylation activity of decitabine is replication-dependent and requires several cell divisions to complete the demethylation of each DNA strand [31], these patients may require multiple treatment cycles to obtain efficient responses [32, 33]. Additionally, a transient 3-day exposure to low-dose DAC has been reported to produce a memory-type antitumor response. In our study, patients were treated with low-dose DAC for five days, and some patients also achieved responses by the end of the follow-up period; therefore, the long-term clinical benefits have yet to be assessed using careful follow-up measurements of the responses to subsequent therapies.

We also identified that there might be a significant correlation between the PFSs following previous treatments and the clinical responses of the low-dose DAC and low-dose DAC combined with CIK groups. Specifically, the patients with long-term PFSs related to previous treatments may have achieved superior curative effects than those with short-term PFSs related to previous treatments.

In our study, the therapeutic efficiencies for liver and spleen malignancies were not obvious. This limitation was possibly due to the high levels of cytidine deaminase activity in these organs; deamination could diminish the concentrations of decitabine to subtherapeutic levels [34]. Whether an inhibitor of cytidine deaminase combined with low-dose DAC could potentially overcome this problem remains to be confirmed.

No obvious effects on some digestive cancers were noted, which may be due to an inadequate number of treatment cycles or because the burdens of the tumors were too large in these patients. This phenomenon is consistent with that of a previously reported clinical study of advanced solid tumor malignancies [35] in which responses were also not observed in patients with esophagus and colon tumors. Future research will require analyses of pretreatment and posttreatment tissue samples to evaluate possible methylation-specific biomarkers and thereby identify patients who will benefit most from the appropriate therapy cycles or pretreatments.

Whether the responses to decitabine are dependent on DNA demethylation is difficult to determine from the present data. Our results indicate that DAC was biologically active as assessed via mRNA reexpression and DNA demethylation in both cell lines and the PBMCs from the patients. The effects of DAC on MAGEA-1, MAGEA-3, and p16 expressions in blood samples and cell lines are consistent with the results of previous studies of a variety of other tumor types [36, 37]. Enhanced expression of MAGEA-1 and MAGEA-3 on the surfaces of malignant cells might lead to more effective “killing” of the tumor cells by cytotoxic T-cell-receptor-based immunotherapeutics. However, p15, which is a gene that is specifically hypermethylated and silenced in hematologic malignancies [38] and has been reported to be demethylated by decitabine, was found to exhibit the reverse response to DAC in the blood sample from UPN 14, and it is possible due to sampling artifact or pharmacodynamics effect. Therefore, we indicated that most of the genes that are differentially expressed during relapse are epigenetically regulated and can be reprogrammed with DAC. These reversals of gene expression perhaps functionally result in enhancements of the chemosensitivity and efficacy of immunotherapy for relapsing and progressing cancer.

In conclusion, we showed that low-dose decitabine-based chemoimmunotherapy had an acceptable safety profile and optimistic activity in patients with various solid and hematological tumors. We provide a new treatment option for patients with refractory advanced tumors. For example, patients who cannot tolerate the side effects of chemotherapy might be treated with low-dose DAC or low-dose DAC combined with adoptive immunotherapy. However, for patients who can tolerate chemotherapy well, low-dose DAC combined with chemotherapy may be a better choice. Furthermore, for low-load tumors, low-dose DAC can be used first and followed by chemotherapy because low-dose DAC may sensitize tumors to chemotherapy and increase the effectiveness of the chemotherapy. Low-dose DAC and chemotherapy could be used simultaneously to treat patients with high-load tumors. Chemotherapy would loosen the tumors and allow DAC to invade tumors easily, which in turn would increase the sensitivity of the tumors to chemotherapy. However, this speculation requires further verification. We believe that low-dose decitabine-based chemoimmunotherapy has a promising future for improving the specificity and efficiency of the treatment of patients with refractory advanced solid tumors.

## Figures and Tables

**Table 1:** Baseline characteristics of patients.

	Decitabine (DAC) (*n* = 8)	DAC combined with chemotherapy (*n* = 18)	DAC combined with CIK (*n* = 5)
Sex			
Male	6 (75%)	10 (55.6%)	5 (100%)
female	2 (25%)	8 (44.4%)	0 (0)
Age (years)	55 (42–77)	57 (28–84)	55 (34–62)
ECOG performance status score			
0-1	7 (87.5%)	16 (88.9%)	5 (100%)
>1	1 (12.5%)	2 (11.1%)	0 (0)
International stage system			
III	2 (25%)	1 (5.6%)	0 (0)
IV	6 (75%)	17 (94.4%)	5 (100%)
Number of previous treatments			
More than two	6 (75%)	18 (100%)	5 (100%)
Number of lesions before DAC treatment			
≤5	3 (37.5%)	6* (33.3%)	4 (80%)
>5	5 (62.5%)	12 (66.7%)	1 (20%)
Primary tumor type			
Gastric cardia adenocarcinoma	2 (25%)	1 (5.6%)	—
Colorectal adenocarcinoma	1 (12.5%)	2 (11.2%)	—
Hepatocellular carcinoma	1 (12.5%)	—	4 (80%)
Intrahepatic bile ducts adenocarcinoma	1 (12.5%)	—	—
Alveolar carcinoma	1 (12.5%)	—	—
Malignant pleural tumors	1 (12.5%)	—	—
Esophageal adenocarcinoma	1 (12.5%)	2 (11.2%)	—
Non-Hodgkin's lymphoma	—	5 (28%)	—
Hodgkin's lymphoma	—	1 (5.6%)	—
Lung adenocarcinoma	—	4 (22.4)	—
Cervical squamous cell carcinomas	—	1 (5.6%)	—
Ovary serous papillary cystadenocarcinoma	—	1 (5.6%)	—
Tubal serous adenocarcinoma	—	1 (5.6%)	—
Pancreatic cancer	—	—	1 (20%)

*Note*. Date is number (%) or media (range). NHL: Non-Hodgkin's lymphoma; ECOG: Eastern Cooperative Oncology Group; *one patient with an increased CA125 level.

**Table 2:** Clinical characteristics of patients.

Subject	Baseline diagnosis	Style of previous therapy	Response to previous therapy	Style of DAC therapy	Response to DAC therapy	PFS to DAC therapy
UPN 1*	Gastric cardia adenocarcinoma	Chemo × 4, TACE × 2	PD	DAC	PD	0
UPN 2	Colorectal adenocarcinoma	Chemo × 10, surgery	PD	DAC	SD	3
UPN 3	Hepatocellular carcinoma	TACE, live transplant	PD	DAC	PD	5
UPN 4*	Gastric cancer	Chemo × 12	PD	DAC	PD	0
UPN 5**	Esophageal adenocarcinoma	Surgery	PD	DAC	SD	5
UPN 6*	Intrahepatic bile ducts adenocarcinoma	Radiation	PD	DAC	PD	2
UPN 7*	Alveolar carcinoma	Chemo × 4, radiation	PD	DAC	PD	1
UPN 8	Malignant pleural tumors	Chemo × 9	PD	DAC	PR	12
UPN 9	Diffuse large B-cell lymphoma	Chemo × 8	PD	DAC + chemo	SD	5
UPN 10*	Non-Hodgkin's lymphoma	Chemo × 5, surgery	PD	DAC + chemo	PD	0
UPN 11*	Non-Hodgkin's lymphoma	Chemo × 25	PD	DAC + chemo	PD	2
UPN 12	Non-Hodgkin's lymphoma	Chemo × 12	PD	DAC + chemo	PD	6
UPN 13*	Non-Hodgkin's lymphoma	Chemo × 9	PD	DAC + chemo	PD	2
UPN 14	Hodgkin's lymphoma	Chemo × 15	PD	DAC + chemo	SD	4
UPN 15	Colon adenocarcinoma	Chemo × 15	PD	DAC + chemo	PD	2
UPN 16	Colorectal cancer	Chemo × 10, gamma knife argon helium knife	PD	DAC + chemo	SD	0
UPN 17	Esophageal squamous cell carcinomas	Chemo × 7, surgery	PD	DAC + chemo	PD	3
UPN 18	Cardia adenocarcinoma	Chemo × 5	PD	DAC + chemo	SD	6
UPN 19**	Small cell carcinoma of the esophagus	Chemo × 8, surgery, and radiation	PD	DAC + chemo	SD	2
UPN 20	Lung adenocarcinoma	Chemo × 3, surgery, and radiation	PD	DAC + chemo	PR	4
UPN 21*	Lung adenocarcinoma	Chemo × 4	PD	DAC + chemo	SD	5
UPN 22*	Lung adenocarcinoma	Chemo × 7	PD	DAC + chemo	SD	4
UPN 23	Lung adenocarcinoma	Chemo × 6, radiation	PD	DAC + chemo	PD	3
UPN 24	Cervical squamous cell carcinomas	Chemo × 10, radiation	PD	DAC + chemo	PD	4
UPN 25	Ovary serous papillary cystadenocarcinoma	Chemo × 28, surgery	PD	DAC + chemo	PR	5
UPN 26	Tubal serous adenocarcinoma	Chemo × 10, surgery	PD	DAC + chemo	SD	7
UPN 27	Pancreatic cancer	Chemo × 9, surgery	PD	DAC + CIK	SD	9
UPN 28	Hepatocellular carcinoma	TACE, radiation	PD	DAC + CIK	SD	5
UPN 29	Hepatocellular carcinoma	Surgery, TACE, and radiation	PD	DAC + CIK	PD	4
UPN 30	Hepatocellular carcinoma	TACE × 6	PD	DAC + CIK	SD	10
UPN 31	Hepatocellular carcinoma	Surgery, TACE × 2	PD	DAC + CIK	SD	8

*Note*. CIK: cytokine induced killer cells; TACE: transcatheter arterial chemoembolization; DAC + chemo: decitabine in combination with chemotherapy; DAC + CIK: decitabine in combination with CIK; *patients died because of disease progression or infection; **patient died from giving up treatment for less than three cycles.

**Table 3:** Summary of adverse events.

Toxicity	DAC		DAC + chemo		DAC + CIK	
	Grades 1-2 *n* (%)	Grades 3-4 *n* (%)	Grades 1-2 *n* (%)	Grade 3-4 *n* (%)	Grades 1-2 *n* (%)	Grades 3-4 *n* (%)
Neutrogena	1 (12.5)	1 (12.5)	10 (55.56)	6 (33.33)	2 (40)	1 (20)
Nausea	0 (0)	0 (0)	5 (27.78)	0 (0)	0 (0)	0 (0)
Fatigue	0 (0)	2 (25)	5 (27.78)	0 (0)	2 (40)	0 (0)
Drowsiness	0 (0)	1 (12.5)	2 (11.11)	0 (0)	0 (0)	0 (0)
Thrombocytopenia	0 (0)	0 (0)	1 (5.56)	0 (0)	0 (0)	0 (0)
Hyperhidrosis	0 (0)	0 (0)	1 (5.56)	0 (0)	0 (0)	0 (0)

*Note.* Data is *n* (%). CIK: cytokine induced killer cells; DAC + chemo: decitabine in combination with chemotherapy; DAC + CIK: decitabine in combination with CIK.

**Table:** (a)

	*n*	Cycles (*n*)	PR *n* (%)	SD *n* (%)	PD *n* (%)	ORR *n* (%)
DAC	8	2	0 (0)	4 (50)	4 (50)	4 (50)
	3	4	0 (0)	1 (33.33)	2 (66.67)	1 (33.33)
	3*	6	1 (50)	0 (0)	1 (50)	1 (50)

DAC + chemo	18	2	1 (5.6)	12 (66.7)	5 (27.8)	13 (72.3)
	11	4	2 (18.2)	4 (36.4)	5 (45.5)	6 (54.5)
	11	6	2 (18.2)	5 (45.5)	4 (36.4)	7 (63.6)

DAC + CIK	5	2	0 (0)	4 (80)	1 (20)	4 (80)
	5	4	0 (0)	4 (80)	1 (20)	4 (80)
	5	6	0 (0)	4 (80)	1 (20)	4 (80)

*Note*. Data is *n* (%). CIK: cytokine induced killer cells; DAC + chemo: decitabine in combination with chemotherapy; DAC + CIK: decitabine in combination with CIK; PR: partial response; SD: stable disease; PD: progressive disease; ORR: objective response rate. Tumors were assessed every 2 cycles by using RECIST 1.0; RECIST: the Response Evaluation Criteria in Solid Tumors; *one patient discontinued treatment attributable to digestive tract hemorrhage.

**Table:** (b)

	Partial response	Stable disease	Progressive disease
DAC	Malignant pleural tumor	Colorectal adenocarcinoma Esophageal adenocarcinoma	Alveolar carcinoma Intrahepatic bile ducts adenocarcinoma Hepatocellular carcinoma Gastric cancer

DAC + chemo	Ovary carcinoma Lung adenocarcinoma	Tubal serous adenocarcinoma Lung adenocarcinoma Cardia adenocarcinoma Hodgkin's lymphoma Non-Hodgkin's lymphoma	Cervical squamous cell carcinomas Lung adenocarcinoma Small cell carcinoma of the esophagus Esophageal squamous cell carcinomas Colon adenocarcinoma Non-Hodgkin's lymphoma

DAC + CIK	—	Pancreatic cancer Hepatocellular carcinoma	Hepatocellular carcinoma

*Note.* CIK: cytokine induced killer cells; DAC + chemo: decitabine in combination with chemotherapy; DAC + CIK: decitabine in combination with CIK.

**Table 5:** Baseline diagnosis of patients whose PFS was significantly prolonged compared to their previous PFS.

	*n*	Baseline diagnosis
DAC	2	Hepatocellular carcinoma
		Esophageal adenocarcinoma

DAC + chemo	10	Hodgkin's lymphoma (3)
		Respiratory tract cancer (2)
		Lung adenocarcinoma (2)
		Urogenital neoplasms (3)

DAC + CIK	3	Pancreatic cancer
		Hepatocellular carcinoma (2)

*Note.* CIK: cytokine induced killer cells; DAC + chemo: decitabine in combination with chemotherapy; DAC + CIK: decitabine in combination with CIK. *P* value < 0.05 was considered significant.

**Table:** (a)

qPCR primer sequence		
RASSF1A	Forward	5′-GCTGAGCGTCACGGCCAAGT-3′ 5′-ATGCTGAAGGCGTCCCAGTT-3′
	Reverse	

MAGEA-1	Forward	5′-TCCGCCTTTCCCACTACCAT-3′ 5′-TCCAGCATTTCTGCCTTTGT-3′
	Reverse	

MAGEA-3	Forward	5′-GGAGTCCGAGTTCCAAGCAG-3′ 5′-AGGCAGGTGGCAAAGATGTA-3′
	Reverse	

P15	Forward	5′-CAACGGAGTCAACCGTTTCGG-3′ 5′-CAGCACCACCAGCGTGTCCAG-3′
	Reverse	

p16	Forward	5′-CTGGACACGCTGGTGGTGCT-3′ 5′-CTATGCGGGCATGGTTACTGC-3′
	Reverse	

BRCA1	Forward	5′-AGAAACCACCAAGGTCCAAA-3′ 5′-CCAAGGGTGAATGATGAAAG-3′
	Reverse	

*β*-Actin	Forward	5′-AAAGACCTGTACGCCAACAC-3′ 5′-GTCATACTCCTGCTTGCTGAT-3′
	Reverse	

**Table:** (b)

Primer sequence		
MAGEA-1 (M)	Forward	5′-AGGAGGGGATAAATATTTGGTTATAC-3′ 5′-GCTCAAATCAATAAAAAAAACGTC-3′
	Reverse	

MAGEA-1 (U)	Forward	5′-AGGGGATAAATATTTGGTTATATGT-3′ 5′-CACTCAAATCAATAAAAAAAACATC-3′
	Reverse	

MAGEA-3 (M)	Forward	5′-GGTAGTATCGTTGTTAGGATGTGAC-3′ 5′-AACCCTCTATCTAAAATAAAACCCG-3′
	Reverse	

MAGEA-3 (U)	Forward	5′-GGTAGTATTGTTGTTAGGATGTGATG-3′ 5′-ACCCTCTATCTAAAATAAAACCCAC-3′
	Reverse	
